# Feasible and effective use of a simulation-based curriculum for post-graduate emergency medicine trainees in India to improve learner self-efficacy, knowledge, and skills

**DOI:** 10.1186/s12245-021-00363-8

**Published:** 2021-07-27

**Authors:** T. Ahluwalia, S. Toy, C. Gutierrez, K. Boggs, K. Douglass

**Affiliations:** 1 Division of Emergency Medicine, Children’s National Medical Center, 111 Michigan Ave NW, Washington DC, 20010 USA; 2 Department of Pediatrics, Children’s National Medical Center, 111 Michigan Ave NW, Washington, DC 20010 USA; 3 Department of Anesthesiology and Critical Care Medicine, Johns Hopkins School of Medicine, Baltimore, USA; 4 George Washington University School of Medicine and Public Health, Washington DC, USA

**Keywords:** Simulation, India, Pediatric emergency medicine, Procedures

## Abstract

**Background:**

Pediatric emergency medicine training is in its infancy in India. Simulation provides an educational avenue to equip trainees with the skills to improve pediatric care. We hypothesized that a simulation-based curriculum can improve Indian post-graduate emergency medicine (EM) trainees’ self-efficacy, knowledge, and skills in pediatric care.

**Methods:**

We designed a simulation-based curriculum for management of common pediatric emergencies including sepsis, trauma, and respiratory illness and pediatric-specific procedures including vascular access and airway skills. Training included didactics, procedural skill stations, and simulation. Measures included a self-efficacy survey, knowledge test, skills checklist, and follow-up survey. Results were analyzed using the Wilcoxon signed-rank test and paired-samples t test. A 6-month follow-up survey was done to evaluate lasting effects of the intervention.

**Results:**

Seventy residents from four academic hospitals in India participated. Trainees reported feeling significantly more confident, after training, in performing procedures, and managing pediatric emergencies (p < 0.001). After the simulation-based curriculum, trainees demonstrated an increase in medical knowledge of 19% (p < 0.01) and improvement in procedural skills from baseline to mastery of 18%, 20%, 16%, and 19% for intubation, bag-valve mask ventilation, intravenous access, and intraosseous access respectively (p < 0.01). At 6-month follow-up, self-efficacy in procedural skills and management of pediatric emergencies improved from baseline.

**Conclusions:**

A simulation-based curriculum is an effective and sustainable way to improve Indian post-graduate EM trainees’ self-efficacy, knowledge, and skills in pediatric emergency care.

## Background

Pediatric mortality in India is high, with an under-five mortality rate of 36.6 per 1000 live births as compared to 6.5 per 1000 live births in the USA [1]. Despite this, the subspecialty of pediatric emergency medicine (PEM) is in its infancy in India. PEM training is essential to equip physicians with knowledge and skills to manage acutely ill children. As emergency medicine (EM) training programs grow in India, progress is being made to enhance education related to management of pediatric emergencies.

Simulation allows trainees to practice skills, especially for low-frequency high impact critical events. Simulation-based training has been more effective than traditional approaches at increasing learners’ satisfaction, knowledge, and skills [2]. With the need for specialized emergency care for children and the limited training availability in India, we have proposed the use of simulation to improve medical knowledge and procedural skills. Our primary objective is to improve Indian post-graduate EM trainees’ self-efficacy, knowledge, and skills in pediatric care with the use of a simulation-based curriculum.

## Methods

### Study setting and population

This study was conducted at 4 academic hospitals in India. All trainees from each site were invited to participate.

### Study design and procedures

The Ronald Reagan Institute of Emergency Medicine at the George Washington University has collaborated with partners in India for 14 years in 3-year post-graduate EM training programs [3, 4]. Training includes didactics, clinical rotations, research, and annual examinations. Pediatric emergency training includes 1–2 months per year of on-site education in India from visiting faculty from the USA, Canada, and UK. The 4 academic sites included in this study have received varying amounts of PEM simulation training based on equipment availability in the past. Our collaboration allowed for the provision of simulation equipment to 4 sites to allow for deliberate PEM training. Based on a needs assessment in 2019, completed by program directors, senior trainees, and visiting PEM faculty, we identified areas with the greatest knowledge gap and developed a corresponding simulation-based curriculum. This study included procedures (intravenous access (IV), intraosseous access (IO), bag-valve mask ventilation (BVM), intubation), and scenarios focused on septic shock, status asthmaticus, and blunt abdominal trauma. Education sessions took place over 2–3 days at each site.

### Educational intervention

Visiting faculty were equipped with tools to lead simulations including video modules on how to conduct simulation; simulation cases from MedEd portal; simulation mannequins and equipment; and faculty support [5, 6]. One of 4 visiting faculty members created the curriculum and it was run by the remaining faculty members at various sites. We used a validated simulation-based mastery learning framework for educational interventions to standardize training [7]. This consists of seven features: baseline testing, clear learning objectives, engagement of an educational activity, identified minimum standards to pass, formative testing, and continued practice until mastery-level achievement [7]. For procedural skills, trainees were provided with checklists of skill competencies (IV, IO, BVM, intubation). Checklists provided increased validity between evaluators by assessing concrete and measurable skills [8]. Participants practiced each skill until they met 100% of objectives (mastery) while being coached and evaluated by visiting faculty who used an evaluation checklist over 1 day.

Trainees were then divided into groups of 4–5 for simulation cases to apply their medical knowledge, as well as, practice their procedural skills. Each group included learners from each training year, with a third-year trainee as team leader. The participants must have been present during procedural skills training. The cases and associated procedures were septic shock (IO), status asthmaticus (IV), and pediatric blunt abdominal trauma (IV/BMV/intubation). After each case, there was a debriefing, followed by a post-questionnaire and post-test.

### Measures and data analysis

We employed systematic training and evaluation guided by the Kirkpatrick 4-step framework to monitor this project’s progress and to evaluate its effectiveness [9] (Table 1). This was a pre-test and post-test design whereby participants served as their own controls. Participants completed a survey measuring their self-efficacy in performing procedures (IV, IO, BVM, and intubation) and in managing pediatric emergencies, respiratory emergencies, trauma, and shock. We also collected demographic information including the number of prior procedures that they completed. Participants completed a knowledge test before and after the educational interventions to evaluate medical management. Additionally, they completed a procedural skills test after didactic training, and after reaching mastery for each skill to evaluate the impact of skills training. Additionally, a random subset of participants performed procedures during simulated cases, which were evaluated by independent raters using a checklist. We calculated percentage scores for the knowledge scores and number of completed procedural steps by dividing each participant’s score by the maximum possible score, and these percentage scores were used in the analyses and reporting. Six months later, participants completed a follow-up survey to assess sustainability of skills and self-efficacy.

**Table 1:** Kirkpatrick 4-step assessment model

Level	Measurement	Measurement in this study
1	Reaction: the degree that participants enjoyed the training	Simulation evaluation forms: pre-questionnaire and post-questionnaire
2	Learning: the degree that participants gained knowledge, skills, and confidence by participating in the study	Skill evaluation forms: pre-test and post-test
3	Behavior: the degree that participants applied what they gained from the training	Skill evaluation forms during simulation and by 6-month follow-up survey
4	Results: the degree that targeted outcomes occur as a result of training	Six-month follow-up survey

The self-report items regarding trainee self-efficacy were on an 11-point scale from 0 to 10. Assumptions for parametric tests were not met for these average self-efficacy scores, as the Shapiro-Wilk’s test was significant. Therefore, we compared these scores using the non-parametric Wilcoxon signed-rank tests. A set of paired samples t tests were used to compare pre-test and post-test scores for knowledge, as well as, for procedural performance scores. All statistical analyses were conducted with Statistical Package for the Social Sciences (IBM SPSS Statistics for Mac, Version 25.0. Armonk, NY:IBM Corp.) with significance level set at *p* < 0.05.

## Results

Seventy EM trainees from four sites in India participated in the study. There were 28 trainees from Madurai (40%), 16 from Kolkata (23%), 16 from Mumbai (23%), and 10 from Bhubaneswar (14%). The majority of participants were males (66%); 11% were in their first year of training, 39% in their second year of training, and 50% in their third year of training (Table 2). Fifty-seven trainees had prior simulation experience with participants having placed up to 10 IVs, 5 IOs, 30 BVM, and 20 intubations. The total number of procedures performed in pediatric practice included 50 IVs, 2 IOs, 100 BMV, and 30 intubations (Table 3).

**Table 2:** Demographics

Variable	Bhubaneswar (*n* = 10)	Kolkata (*n* = 16)	Madurai (*n* = 28)	Mumbai (*n* = 16)	Overall (*n* = 70)
Gender, n (%)					
Female	4 (40%)	5 (31%)	8 (27%)	7 (44%)	24 (34%)
Male	6 (60%)	11 (69)	20 (71%)	9 (56%)	46 (66%)
Prior training, n (%)					
No	9 (90)	15 (94%)	24 (86%)	16 (100%)	64 (91%)
Yes	1 (10%)	1 (6%)	4 (14%)	–	6 (9%)
Training year, n (%)					
1	–	1 (6%)	7 (25%)	–	8 (11%)
2	4 (40%)	7 (44%)	10 (36%)	6 (37.5%)	27 (39%)
3	6 (60%)	8 (50%)	11 (39%)	10 (62.5%)	35 (50%)

**Table 3:** Previous experience with procedures

Procedure	Median	Minimum	Maximum	Range
# IV SIM	0	0	10	10
# IV Real Patient	4	0	50	50
# IO Sim	0	0	5	5
# IO Real Patient	0	0	2	2
# BVM Sim	2	0	30	30
# BVM Real Pt	3	0	100	100
# Intubate Sim	0	0	20	20
# Intubate Real Patient	0	0	30	30

Participants’ felt significantly more confident, after training, in performing procedures and managing pediatric emergencies (p < 0.001) (Table 4). Overall medical knowledge (all tests combined) increased from 61 to 80% (p < 0.01). A set of paired samples t tests indicated statistically significant improvement in knowledge scores for sepsis (pre-mean = 58%, SD = 23% vs post-mean = 80%, SD = 17%), t(58) = − 7.27, P < .001); and respiratory issues (pre-mean = 59%, SD = 24% vs post-mean = 81%, SD = 18%), t(58) = − 6.07, P < .001). Trauma knowledge also improved significantly, though with a much smaller magnitude, from the mean of 68% (SD = 29%) to 75% (SD = 24%) (t(58) = − 2.10, p = 0.040).

**Table 4:** Self-reported self-efficacy

Variable	Baseline		Post-test		*z* value	*p* value
	Mean (SD)	Median	Mean (SD)	Median		
Overall self-efficacy in performing included procedures	4.26 (2.10)	4.13	8.59 (1.00)	8.75	− 6.794	< 0.001
Overall self-efficacy in managing included pediatric emergencies	4.69 (1.95)	4.88	8.13 (1.05)	8.00	− 6.793	< 0.001

Mean, standard deviation, median, and Wilcoxon signed-ranks test results comparing overall self-efficacy between pretest and post-test scores in performing included procedures and managing pediatric emergencies (*n* = 61)

Procedural skills from baseline to mastery increased by 18%, 20%, 16%, and 19% for intubation, BVM, IV, and IO, respectively (p < 0.01) (Table 5). Participants’ procedural performance overall was better during the simulation compared to baseline scores except for IO skills, which decreased from 87 to 83% during simulation. Only significant skill gain was observed for BVM from baseline to simulation performance (baseline = 79%, SD = 21% vs sim-mean = 91%, SD = 13%), t(7) = − 3.02, P = .019). Please note the sample sizes are much smaller for these since we were not able to have all participants complete procedures during actual simulations.

**Table 5:** Summary statistics for procedural skills at baseline and mastery and paired samples t test results

Variable	N	Mean (SD)	Min	Max	*t*	*p* value
# of intubation attempts for mastery	55	1.76 (0.79)	1	5		–
Baseline # of completed intubation steps	56	9.79 (2.76)	0	12		–
Baseline % completed intubation steps	56	82% (23%)	0%	100%	− 6.00	< 0.001
Mastery % completed intubation steps	56	100%	100%	100%		
# of BVM attempts for mastery	56	1.64 (0.72)	1	4		–
Baseline # of completed BVM steps	56	4.82 (1.35)	1	6		–
Baseline % completed BVM steps	56	80% (22%)	17%	100%	− 6.53	< 0.001
Mastery % completed intubation steps	56	100%	100%	100%		
# of IV attempts for mastery	54	2.02 (1.54)	1	10		–
Baseline # of completed IV steps	55	5.87 (1.26)	0	7		–
Baseline % completed IV steps	55	84% (18%)	0%	100%	− 6.62	< 0.001
Mastery % completed intubation steps	55	100%	100%	100%		
# of IO attempts for mastery	53	2.19 (0.79)	1	4		–
Baseline # of completed IO steps	55	10.55 (2.56)	0	13		–
Baseline % completed IO steps	55	81% (20%)	0%	100%	− 7.11	< 0.001
Mastery % completed intubation steps	55	100%	100%	100%		

Six months after the educational intervention, participants completed a follow-up survey. Results found that participants placed up to 20 IVs in pediatric simulations and up to 50 IVs in patients with an average self-efficacy of 7.18/10. Participants placed up to 10 IOs in pediatric simulations and up to 3 in patients, with an average self-efficacy of 5.5/10. Participants performed BVM in up to 30 simulated pediatric cases, and up to 30 patients, with an average self-efficacy of 8.75/10. Participants intubated up to 12 pediatric simulated patients and up to 30 patients, with an average self-efficacy of 6.92/10. Average self-efficacy for management of procedures and pediatric emergencies were 7.01 and 7.39, a sustained improvement over pre-intervention self-efficacy scores.

## Discussion

We found that participants had the most experience with BVM and the least experience placing IOs in children. Procedural skills improved the most for BVM, which is likely due to targeted education on pediatric anatomical differences compared to adults. Airway and IV skills improved from baseline as compared to performance during simulation. Based on the follow-up survey, participants had the least self-confidence placing IOs. Further IO training would be useful. Overall, self-efficacy for management of pediatric emergencies improved after the intervention and sustained over time.

Interestingly, procedures were performed more clinically compared to a simulated environment except for IOs, which were only performed on 2 patients at baseline. In contrast, US trainees often practice procedural skills during simulation. Compared to previous studies implementing simulation in a low-resource environment, we found similar improvements in self-efficacy along with post-test score improvement [10].

Our simulation-based curriculum was an effective way to advance trainees self-efficacy, knowledge, and skills with pediatric procedures. We adapted cases based on the Indian context for this study. Further work to improve cultural competency around cases may also improve effectiveness. In this project, visiting faculty conducted the simulation program, and in the future, expanding this curriculum to a broader scale and developing projects focused on capacity building through faculty development among in-country partners may foster a sustainable future for PEM in India.

### Limitations

Our ability to install a sustained intervention was limited due to time constraints of visiting faculty in India. This study was also limited by low interrater reliability as we had different visiting faculty at various sites. We reported the number of procedures, as well as, self-efficacy in performing procedures and in management of pediatric emergencies at baseline, during, and at 6-month follow-up. However, it is difficult to align the 6-month follow-up directly with the study. Although we designed this study on Kirkpatrick’s 4-step model, we were unable to assess outcomes on clinical care. Future studies on the translation to clinical care and patient outcomes would be valuable.

## Conclusion

A simulation-based curriculum is an effective and sustainable way to improve Indian post-graduate EM trainees’ self-efficacy, knowledge, and skills in pediatric emergency care.

## Data Availability

The datasets used and/or analyzed during the current study are available from the corresponding author on reasonable request.

## Abbreviations

BVM: Bag-valve mask ventilation
EM: Emergency medicine
IV: Intravenous access
IO: Intraosseous access
PEM: Pediatric emergency medicine
